# Supplementary material for: Intersectional stigma and adherence to antiretroviral (ART) among sexual minority men (SMM) living with HIV in Nigeria: A Qualitative Inquiry
Source: PLoS One. 2025 Dec 29;20(12):e0338933. doi: 10.1371/journal.pone.0338933 (PMC12747391; doi:10.1371/journal.pone.0338933)
Supplement: Attachment — Submitted filename: PONE-D-24-43676_R2-65-73.docx [file pone.0338933.s005.docx]

**Editors Comments**

| **Comments** | **Response** | **2nd round of comments** | **Author Response to 2nd round comments** | **3^rd^ rounf of comments** |
| --- | --- | --- | --- | --- |
| Please ensure that page numbers and/or line numbers are correctly  indicated. | This has now been completed. | OK |  | OK |
| Define the acronym "HCP" to improve clarity for readers  unfamiliar with this terminology | This has now been completed. | OK, please be precise that you mention abstract | The acronym ‘HCP’ was first utilized in the methods (line 86) and has now been defined when first used and only the acronym utilized afterwards.  Healthcare provider was spelled out in the abstract and since it was utilized only once, we didn’t utilize the  acronym. | OK |
| Clarify the distinction between "sexual minority men (SMM)" and "men who have sex with men (MSM)" to ensure conceptual precision. | We opted to utilize the term SMM rather than MSM due to SMM being fully encompassing the sexual orientation and identity of gay, bisexual, and other sexual minority men and MSM reducing their identities to only sexual activity. The distinction has  now been made in the revised manuscript. | OK, please mention the sentence and where in the manuscript.  Introduction ? Discussion ? | The acronym SMM (sexual minority men) was first utilized in the abstract (line 21) and was defined there and the acronym utilized afterwards.  Additionally, SMM was first utilized in the introduction and was first defined then (Line 47-48) and the acronym utilized afterwards. | OK |
| In the statement, "Additionally, SMM living with HIV in Nigeria experience suboptimal outcomes on the HIV care continuum,"  specify whether this comparison is | This was meant to be in comparison to the general population and more elaboration has now been provided on this point. | Please mention the sentence and where in the manuscript (section, line, etc.)!  About this sentence, line 50 in the introduction section :  “Additionally, compared to the general population, | Statistics demonstrating disparities in HIV outcomes among SMM compared to the general population has now been added to lines 55 to 59 | Thank you for adding these statistics. Now please add the statistics for the general population to which you are comparing them. |

| being made against other countries or  different population groups. |  | SMM living with HIV in Nigeria experience suboptimal outcomes on the HIV care continuum (diagnosis of HIV infection, linkage to HIV care, receipt of HIV care, retention in HIV care, and achievement and maintenance of HIV viral suppression). |  |  |
| --- | --- | --- | --- | --- |
|  |  | Please add few numbers and details about these ideas. |  |  |
| The exclusion of transgender women is justified based on differing lived experiences. However, please provide a more precise explanation of these differences and their implications for the study. | We have now elaborated on how the lived experiences of transgender women might differ from that of SMM to justify their non-inclusion in our sample. | Please, when you copy and paste, copy all the sentence (with “T” at the beginning)  +  Please mention the sentence and where in the manuscript .  You added this sentence at line 97 in the method section (I needed to find where in the manuscript) : “Specifically, transgender women might have experiences related to their gender expression, presentation, and conformity (or lack thereof) to societal expectations of gender performance, that cisgender SMM don’t experience.” | We have now cited published literature that has called for differentiation in research for SMM and transgender populations in lines 100-102 | OK |
|  |  | Please be more specific with example and numbers. |  |  |

| The manuscript states that Excel was used for data coding. Was no other qualitative data analysis software employed?  Additionally, clarify whether coding  was cross- validated between researchers to ensure rigor. | We initially utilized Excel to organize the transcripts and then utilized NVIVO to conduct comprehensive coding of all transcripts. This was cross-validated by two researchers on our team. This information has now been included in the revised manuscript. | OK, please mention the sentence and where in the manuscript | This was included in lines 128-130 | OK, don't forget to add the versions of Excel and NVivo used. |
| --- | --- | --- | --- | --- |
| Provide a clear definition of "community" as used in the study. | In this study, community stigma was defined as stigma emanating from the physical community/neighborhood where participants lived. This has now been elucidated  in the revised manuscript. | Please mention the sentence and where in the manuscript.  My comment was relative to  “community” and not “community stigma” as asked in the next comment.  It’s still not very clear, sometimes you mention community without definition :  Line 101 “community- based organization (CBO)” : I supposed relative to SMM  community ? “  Line 512 “Our findings underscore the importance of developing  community informed”  : Still SMM community  If it is only SMM community, please define it. | We assume the context in which the term “community” is used would lend itself to the definition of the term. We have now denoted what we mean by community in each instance of the word being utilized, especially when describing the SMM community specifically to avoid any confusion. You can find this in lines 39, 458, and 515 | Ok thank you it’s better now |
| Elaborate on the distinction between "community stigma" and "enacted stigma" to  enhance conceptual clarity. | We have now provided brief descriptions for each domain of stigma discussed in this manuscript | OK, please mention the sentence and where in the manuscript. | Please find the definition for enacted stigma in lines 160- 161 and for  community stigma in line184) | OK |

| The assertion that "the illegality of homosexual relationships in Nigeria could be the primary reason for this  phenomenon" would be more appropriately placed in the  Introduction. | We agreed and have now removed this from the results and included it in the introduction to better frame the impetus for the paper. | I don’t find it sorry | Please find in lines 67- 69 | OK, please add the associated reference. |
| --- | --- | --- | --- | --- |

| In the statement, "A vast majority of the HCPs and some SMM noted that stigmatization had a negative impact on engagement in HIV care," please specify the number or percentage of participants who expressed this view. In a broader way, specify in the entire results section if several individuals are concerned for a result  and define it clearly. | We have now made this suggested edit and quantified the saliences of the themes being discussed in the results section | I’m not sure to understand how do you use “several”, for example at line 158 “several (<50%)” is not clear.  Please be more specific, for example indicate number of people who expressed this on number of people who expressed (n/N) or don’t use this unclear word | We have now removed all quantification of the qualitative findings, as it defeats of purpose of conducting qualitative research.  We have simply presented the most salient themes from the interviews without any quantitative quantification. | OK |
| --- | --- | --- | --- | --- |
| Clarify whether respondents provided both negative and positive perspectives or whether different individuals contributed distinct  viewpoints at different times. | Participants usually noted both negative and positive perspectives and we have merely provided representative quotes to illustrate the large points being made | Ok thank you |  | OK |

| Address the potential impact of  participant compensation on study findings. | We have now included this in the discussion section | Please mention the sentence and where in the manuscript.  Again I need to copy and paste line 506 in the discussion section  :  “There is always a potential that study compensation could influence study findings but given the minimal compensation provided for study  participation, we don’t believe that is a major cause for concern in the current study”  This is not clear, how do you know that 20 dollars could not be influence these results ? How did you do to minimize this bias ? “Lastly, we did not review objective measurements of engagement in HIV care including laboratory results and provider medical notes, limiting our ability to explore our intersectional stigma might influence HIV care outcomes beyond anecdotal accounts from the study participants.” It’s not clear, please rephrase it. | We agree that study compensation has the potential to influence study findings. This is understood within the field of empirical research beyond public health. We also believe in the core tenets of community-based participatory research, which outlines the needs to properly compensate individuals for their time and efforts. The amount of $20 was arrived at based on consulation with community advisory boards for previous studied and our understanding of the community we have worked with for over 10 years. We cannot say with certainty that the compensation was not a major motivator of research participation, no study can state that as a fact. There is also no way to “minimize this bias” as we believe people should be compensated for their time and efforts and we did so accordingly. | Yes, I understand. I was just asking to discuss this point. Please feel free to incorporate your response appropriately into your manuscript. |
| --- | --- | --- | --- | --- |
|  |  |  | The sentence about not reviewing lab and medical notes has now been reviewed for easier readability. Find in lines 512-514 |  |
| Expand the  discussion on the implications of the | This has now been expounded on. | Ok, please mention  the sentence and where in the | Find in line 413-427 | OK |

| illegality of  homosexuality in Nigeria. |  | manuscript. |  |  |
| --- | --- | --- | --- | --- |
| The following sentence is lengthy and somewhat unclear: "It is important to intentionally engage both SMM and relevant stakeholders in the designing and implementation of these interventions and the utilization of a strengths- based and resilience-focused [44], rather than a deficit, approach and incorporation of key tenets of community-based participatory research are paramount to ensuring these interventions are acceptable and utilized by the target population." Please consider rewording for clarity and  conciseness. | This has now been edited for brevity | I don’t find it sorry | Find the edited version in lines 494-496 | OK |
| Include additional discussion on study limitations to provide a more comprehensive evaluation of its  scope and constraints. |  | Not adressed | We have provided a comprehensive list of study limitations in lines 502-518. | OK |
