# Supplementary material for: Intersectional stigma and adherence to antiretroviral (ART) among sexual minority men (SMM) living with HIV in Nigeria: A Qualitative Inquiry
Source: PLoS One. 2025 Dec 29;20(12):e0338933. doi: 10.1371/journal.pone.0338933 (PMC12747391; doi:10.1371/journal.pone.0338933)
Supplement: Attachment — Submitted filename: Response to Reviewer Comments.docx [file pone.0338933.s002.docx]

April 30, 2025

Emily Chenette, PhD

Editor-in-Chief

PLOS ONE

Dear Dr. Chenette,

We are pleased with the opportunity to resubmit our manuscript, *Intersectional stigma and adherence to antiretroviral (ART) among sexual minority men (SMM) living with HIV in Nigeria: A Qualitative Inquiry, in* consideration for publication as a research article in *PLOS ONE.* We believe we have addressed all points from the prior review and that the manuscript, in its’ current state is ready for publication.

We appreciated the thoughtful comments of the reviewers from our initial submission

On the page that follow, we include a table that outlines the reviewer comments and our corresponding responses. In the revised manuscript, changes are highlighted.

Please do not hesitate to contact me if you have any questions or require further information. Thank you for your consideration and we look forward to your response.

**Editors Comments**

| **Comments** | **Response** |
| --- | --- |
| Please ensure that page numbers and/or line numbers are correctly indicated. | This has now been completed. |
| Define the acronym "HCP" to improve clarity for readers unfamiliar with this terminology | This has now been completed. |
| Clarify the distinction between "sexual minority men (SMM)" and "men who have sex with men (MSM)" to ensure conceptual precision. | We opted to utilize the term SMM rather than MSM due to SMM being fully encompassing the sexual orientation and identity of gay, bisexual, and other sexual minority men and MSM reducing their identities to only sexual activity. The distinction has now been made in the revised manuscript. |
| In the statement, "Additionally, SMM living with HIV in Nigeria experience suboptimal outcomes on the HIV care continuum," specify whether this comparison is being made against other countries or different population groups. | This was meant to be in comparison to the general population and more elaboration has now been provided on this point. |
| he exclusion of transgender women is justified based on differing lived experiences. However, please provide a more precise explanation of these differences and their implications for the study. | We have now elaborated on how the lived experiences of transgender women might differ from that of SMM to justify their non-inclusion in our sample. |
| The manuscript states that Excel was used for data coding. Was no other qualitative data analysis software employed? Additionally, clarify whether coding was cross-validated between researchers to ensure rigor. | We initially utilized Excel to organize the transcripts and then utilized NVIVO to conduct comprehensive coding of all transcripts. This was cross-validated by two researchers on our team. This information has now been included in the revised manuscript. |
| Provide a clear definition of "community" as used in the study. | In this study, community stigma was defined as stigma emanating from the physical community/neighborhood where participants lived. This has now been elucidated in the revised manuscript. |
| Elaborate on the distinction between "community stigma" and "enacted stigma" to enhance conceptual clarity. | We have now provided brief descriptions for each domain of stigma discussed in this manuscript |
| The assertion that "the illegality of homosexual relationships in Nigeria could be the primary reason for this phenomenon" would be more appropriately placed in the Introduction. | We agreed and have now removed this from the results and included it in the introduction to better frame the impetus for the paper. |
| In the statement, "A vast majority of the HCPs and some SMM noted that stigmatization had a negative impact on engagement in HIV care," please specify the number or percentage of participants who expressed this view. In a broader way, specify in the entire results section if several individuals are concerned for a result and define it clearly. | We have now made this suggested edit and quantified the saliences of the themes being discussed in the results section |
| Clarify whether respondents provided both negative and positive perspectives or whether different individuals contributed distinct viewpoints at different times. | Participants usually noted both negative and positive perspectives and we have merely provided representative quotes to illustrate the large points being made |
| Address the potential impact of participant compensation on study findings. | We have now included this in the discussion section |
| Expand the discussion on the implications of the illegality of homosexuality in Nigeria. | This has now been expounded on. |
| The following sentence is lengthy and somewhat unclear: "It is important to intentionally engage both SMM and relevant stakeholders in the designing and implementation of these interventions and the utilization of a strengths-based and resilience-focused [44], rather than a deficit, approach and incorporation of key tenets of community-based participatory research are paramount to ensuring these interventions are acceptable and utilized by the target population." Please consider rewording for clarity and conciseness. | This has now been edited for brevity |
| Include additional discussion on study limitations to provide a more comprehensive evaluation of its scope and constraints. |  |
